# Coracoid Impingement and Morphology Is Associated with Fatty Infiltration and Rotator Cuff Tears

**DOI:** 10.3390/jcm11092661

**Published:** 2022-05-09

**Authors:** Saadiq F. El-Amin, Nicola Maffulli, Matthew C. Mai, Hugo C. Rodriguez, Victoria Jaso, Dylan Cannon, Ashim Gupta

**Affiliations:** 1 El-Amin Orthopaedic and Sports Medicine Institute, Lawrenceville, GA 30043, USA; 2 Regenerative Sports Medicine, Lawrenceville, GA 30043, USA; 3 BioIntegrate, Lawrenceville, GA 30043, USA; ashim6786@gmail.com; 4 Department of Musculoskeletal Disorders, School of Medicine and Surgery, University of Salerno, 84084 Fisciano, Italy; n.maffulli@qmul.ac.uk; 5 San Giovanni di Dio e Ruggi D’Aragona Hospital “Clinica Orthopedica” Department, Hospital of Salerno, 84124 Salerno, Italy; 6 Barts and the London School of Medicine and Dentistry, Centre for Sports and Exercise Medicine, Queen Mary University of London, London E1 4DG, UK; 7 School of Pharmacy and Bioengineering, Keele University School of Medicine, Stoke on Trent ST5 5BG, UK; 8 Florida Bone & Joint Specialists, Gulf Breeze, FL 32561, USA; mmai@flboneandjoint.com; 9 Holy Cross Orthopedic Institute, Fort Lauderdale, FL 33334, USA; hcrodrig2112@gmail.com (H.C.R.); dylancannon223@gmail.com (D.C.); 10 Larkin Community Hospital, South Miami, FL 33143, USA; 11 Ross University School of Medicine, Miramar, FL 33156, USA; victoriajaso@mail.rossmed.edu; 12 Future Biologics, Lawrenceville, GA 30043, USA; 13 South Texas Orthopedic Research Institute (STORI Inc.), Laredo, TX 78045, USA; 14 Veterans in Pain (V.I.P.), Valencia, CA 91354, USA

**Keywords:** anterosuperior cuff tear, coracoid morphology, supraspinatus tear, magnetic resonance imaging, rotator cuff fatty infiltration, coracoid impingement

## Abstract

This study describes measurements between the coracoid, glenoid, and humerus; characterizes coracoid shape, rotator cuff fatty infiltration, and quantitatively evaluates coracoid impingement and its association with anterosuperior rotator cuff tears (ASCT). 193 shoulder magnetic resonance imaging (MRI) scans demonstrating: rotator cuff tear; isolated tear of the supraspinatus; tear of supraspinatus and subscapularis, were included. MRI measurements included coracohumeral interval (CHI), coracoid overlap (CO), coracoid recess (CR), coracoglenoid angle (CGA), and coracoglenoid interval (CGI) on axial slices; acromiohumeral interval (AHI) on coronal slices; and coracohumeral interval (CHI) and coracoacromial ligament (CAL) thickness on sagittal slices. The coracoid shape was classified as flat, curved, or hooked. An Independent T-test was used to compare the MRI measurements and the different rotator cuff tear groups. In 79% of the patients with ASCT tears, the coracoid was curved. Axial CHI, CGA, sagittal CHI, and AHI were decreased in ASCT when compared to no tears and isolated supraspinatus tears (*p* < 0.05). CO was increased in ASCT compared to no tears and isolated supraspinatus tears (*p* < 0.05). Patients with an ASCT had a significantly increased subscapularis and supraspinatus Goutallier fatty infiltration score when compared to no tear and isolated supraspinatus tears (*p* < 0.05). These quantitative measurements may be useful in identifying patients at risk for ASCT. Level of Evidence III.

## 1. Introduction

Sub-Coracoid impingement syndrome is caused by compression of the anterior soft tissues of the shoulder between the coracoid process and the humerus [1]. This was described first by Goldthwait in 1909 and defined more precisely by Gerber et al. who described sub-coracoid impingement as a cause of anterosuperior rotator cuff (RC) pathology [1,2]. Following Gerber et al. description, several studies have supported the role of coracoid impingement in anterior shoulder pathology [3,4,5,6,7,8]. Sub-Coracoid impingement affects patients who engage in repetitive shoulder flexion, combined with internal rotation and adduction, such as boxers, martial artists, and baseball and cricket players [9]. The condition can also be seen in patients who engage in repetitive overhead movements such as manual workers, baseball pitchers, and tennis players [10]. Regardless of the exact etiology, patients often complain of anterior shoulder pain with elevation, internal rotation, and abduction at 120–130 degrees of flexion, along with positive coracoid impingement and Yocum’s test [11,12]. Treatment options range from non-surgical options (NSAIDs, injection therapy, and physical therapy) to surgical procedures such as a coracoplasty to decompress the sub-coracoid space [5,7,13,14]. Subsequently, several authors have described both open and arthroscopic approaches for coracoid impingement [15,16,17,18,19]. Fatty atrophy of the RC and progression to RC tears was assessed using CT scans of the shoulder to grade fatty infiltration in RC tears scheduled for surgical repair [20]. Subsequent studies have suggested that progressive fatty infiltration of rotator cuff tendons may have poorer functional outcomes following repair and that fatty infiltration may not be reversed after RC repair [21,22].

The relationship of the coracoid to the glenoid and humerus is highly variable and is dependent on the morphology of the coracoid and the anatomy of the coracoacromial arch. The relationship of the anterior shoulder structures has been described in several cadaver studies [23,24,25,26]. In addition, imaging parameters have been described on computed tomography (CT) [25,27,28], magnetic resonance imaging (MRI) [29,30,31,32,33], single-photon emission computerized tomography scans [34], and ultrasound [35]. Studies have retrospectively assessed limited preoperative MRI parameters after a clinical diagnosis of coracoid impingement and subsequent surgical confirmation was made [31,33].

The primary objective of the present study was to use quantitative MRI parameters of the coracoid and anterior shoulder morphology, and the presence of cuff atrophy, to examine their association with anterosuperior rotator cuff tears (ASCT) and supraspinatus tears, aiming to study the relationship between ASCT and morphometric changes in the above structures. In addition, a qualitative classification of coracoid morphology was used to aid in the identification of coracoid impingement. We hypothesized that several quantifiable measurements of subcoracoid impingement on MRI, and a hook coracoid morphology is associated with increased fatty infiltration and subsequently increased presence of ASCT.

## 2. Materials and Methods

**Figure 1:**
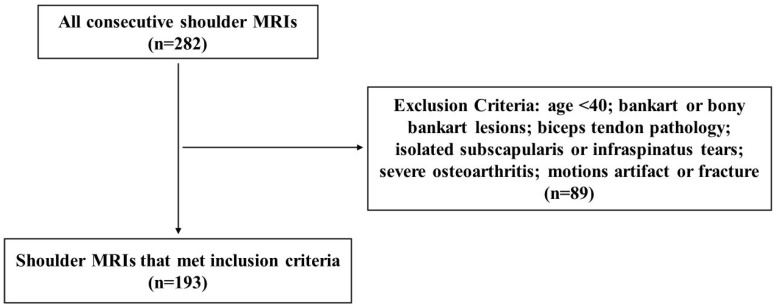
Flow chart indicating the inclusion of MRI images.

Investigational review board approval was obtained by the Southern Illinois University School of Medicine’s Committee (Springfield, IL, USA) for Research Involving Human Subjects for this study. Two hundred and eighty-two consecutive shoulder MRIs from our Hospital (Memorial Medical Center, Springfield, IL, USA) Imaging Database were retrospectively reviewed for this descriptive study. An MRI with a 3.0 Tesla magnet and shoulder coil was used to obtain axial T2 weighted and gradient echo sequence (GRE), oblique coronal T2 weighted, proton density-weighted (PD), oblique sagittal T2 weighted, and PD views in neutral shoulder rotation, with a slice thickness of 3 mm. Exclusion criteria included patients under the age of 40 years old, isolated Bankart or boney Bankart lesions, biceps tendon pathology, isolated subscapularis tear, isolated infraspinatus tear, severe osteoarthritis, and motion artifact, or fracture. One hundred and ninety-three MRIs met the final inclusion criteria (patients above the age of 40, with no evidence of isolated Bankart or bony Bankart lesions, no biceps tendon pathology, no isolated subscapularis tear, and no isolated infraspinatus tear) and were included for analysis (Figure 1).

**Figure 2:**
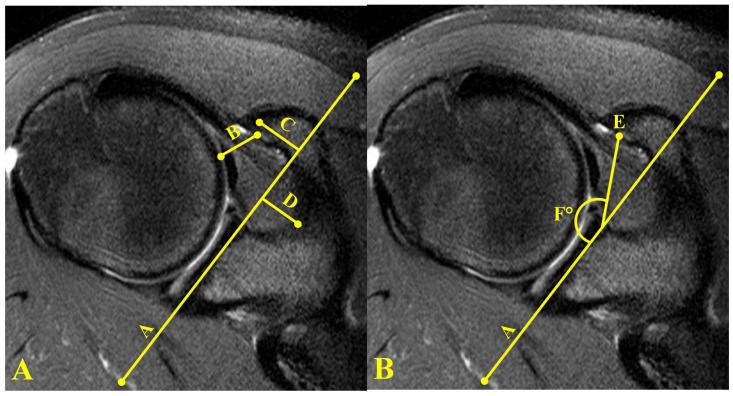
(**A**) Line A: the reference line drawn parallel to the glenoid on axial MRI of the shoulder. Line B: coracohumeral distance measured as the closest distance between coracoid and humerus with a line parallel to line A. Line C: coracoid overlap. Line D: coracoid recess. (**B**) Axial MRI of shoulder showing coracohumeral interval, Line E, and coracoglenoid angle (Angle F).

Quantitative MRI measurements were performed using the Carestream KODAK imaging system (Rochester, NY, USA). Axial MRI measurements included coracohumeral interval (CHI), coracoid overlap (CO), coracoid recess (CR), coracoglenoid angle (CGA), and coracoglenoid interval (CGI) (Figure 2). For the axial measurements, the slice demonstrating the largest area of coracoid was identified. A reference line was first drawn parallel to the glenoid (Line A, Figure 2). The smallest distance between the coracoid and the humeral head was defined as the CHI (Line B, Figure 2A). A line drawn from the tip of the coracoid and bisecting the coracoid perpendicular to line A was defined as the CO (Line C, Figure 2). The coracoid recess was measured from a line drawn perpendicular to line A to the base of the scapula in the area between the glenoid and coracoid (Line D Figure 2). Line E (Figure 2B) represents the CGI and is defined as the line connecting the most anterior point on the glenoid reference line and the coracoid point along with the CHI. The coracoglenoid angle is measured on an axial MRI slice showing both the glenoid face and the coracoid process. The angle is between Line A of the glenoid, and a line to the tip of the coracoid (Angle F, Figure 2B).

**Figure 3:**
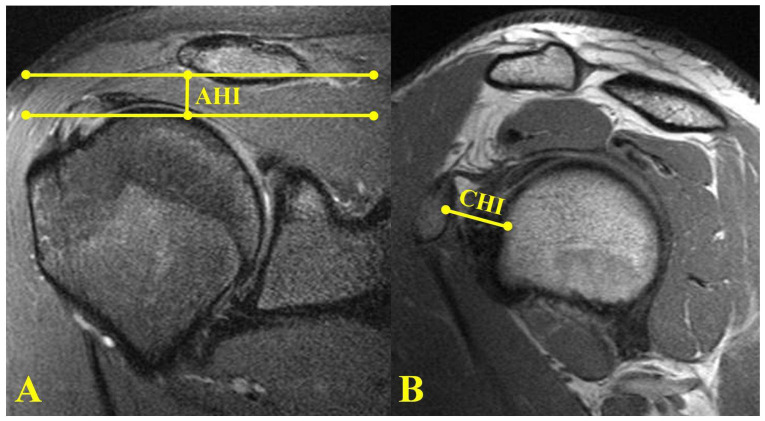
(**A**) Coronal MRI of shoulder showing measurement of coronal acromiohumeral interval (AHI). (**B**) Sagittal MRI of shoulder showing coracohumeral interval (CHI).

**Figure 4:**
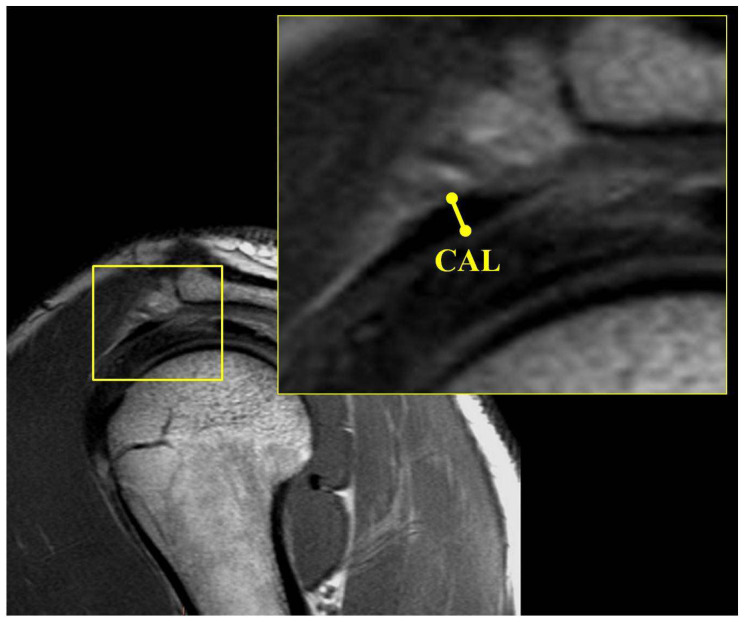
Sagittal MRI of shoulder showing measurement of coracoacromial ligament (CAL).

Coronal MRI measurements included the acromiohumeral interval (AHI). The AHI was measured from a line drawn from the subchondral bone of the center of the humeral head to the subchondral bone of the acromion. This measurement was performed at the coronal slice with the narrowest interval (Figure 3A). Sagittal MRI measurements included the coracohumeral interval (CHI) and the coracoacromial ligament (CAL) thickness. The CHI on the sagittal view was measured as the shortest distance in a line drawn from the subchondral cortex of the humerus to the coracoid (Figure 3B). The coracoacromial ligament was identified in the sagittal plane, and its size was measured at its thickest point (Figure 4).

**Figure 5:**
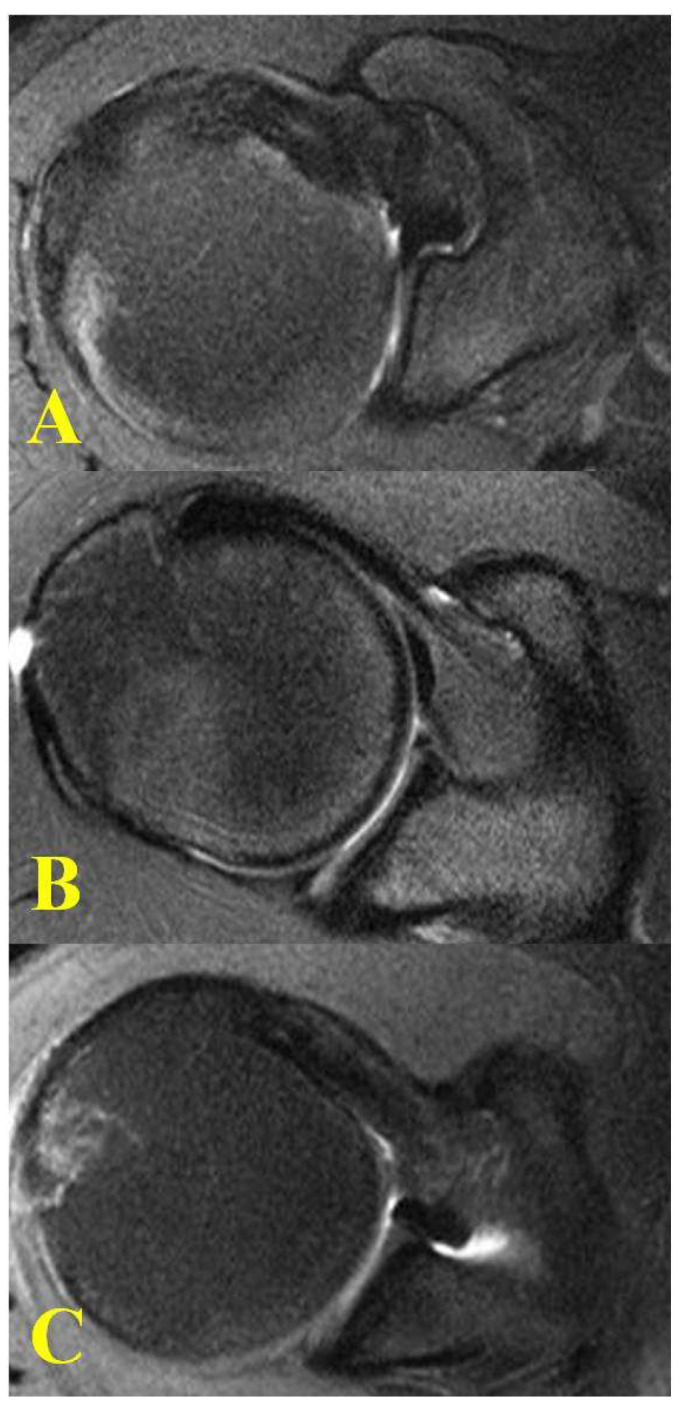
Coracoid Morphology on axial MRI of shoulder: (**A**) Curved; (**B**) Hook; (**C**) Flat.

The presence of rotator cuff fatty infiltration was determined as described by Goutallier et al. [20]. Grade 0 was a normal muscle, Grade 1 was the presence of some fatty streaks, Grade 2 was more muscle than fat, Grade 3 was equal amounts of muscle and fat, and Grade 4 was more fat than muscle. Each coracoid was qualitatively determined as flat, curved, or hooked (Figure 5) [36]. All three MRI slices (Axial/Coronal/Sagittal) were used to determine the appropriate coracoid morphology. The MRIs were reviewed on a dedicated workstation by two Orthopaedic surgery residents (JB, RB) who evaluated the Goutallier classification of fatty infiltration and morphology of the coracoid with absolute consensus for each patient. Three specific groups were defined and included no tear, supraspinatus tear, and ASCT. Descriptive statistics for each group and Independent T-Tests with a statistical significance of *p* < 0.05 were used for analysis. SPSS version 18 (SPSS Inc., Chicago, IL, USA) was used for all statistical analyses.

## 3. Results

The age and sex for each RC tear group are listed in Table 1. The study group included 102 (52.8%) males and 91 (47.2%) females. The average age of the entire cohort was 56 ± 9.9 years (40–88). 100 right shoulders and 93 left shoulders were evaluated. 63 patients had no RC tear, 72 had an isolated supraspinatus tear and 58 had an ASCT. 15 coracoids were classified as flat, 122 as curved, and 56 as hooked. There was a statistically significant increase in mean age in the ASCT group (60.3 ± 10.01) compared with the no-tear group (52.2 ± 9.56) and the supraspinatus group (54.9 ± 8.76) (*p* < 0.05). A curved coracoid was the most common morphology across all tear groups but was significantly higher in isolated supraspinatus tears (58%) and ASCT (79%) (*p* < 0.05).

**Figure 6:**
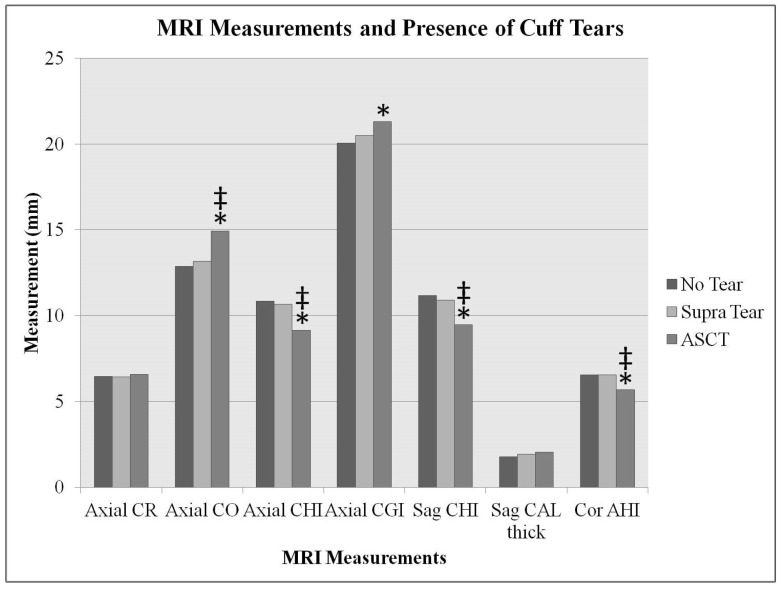
Quantitative MRI measurements in relation to the presence of tears. CR = coracoid recess, CO = coracoid overlap, CHI = coracohumeral interval, CGI = coracoglenoid interval, CAL = coracohumeral thickness and AHI = acromiohumeral interval. * demonstrates statistically significant difference (*p* < 0.05) between ASCT vs no tear group; ‡ demonstrates statistically significant (*p* < 0.05) difference between ASCT vs supraspinatus group.

The mean ± standard deviation (range) for each RC tear group MRI measurement is listed in Table 2. When comparing the ASCT group to the no tear group, the CO and CGI were significantly increased and the axial CHI, CGA, sagittal CHI, and AHI were significantly decreased (*p* < 0.05). When comparing the ASCT group to the supraspinatus group, the CO was significantly increased and the axial CHI, CGA, sagittal CHI, and AHI were significantly decreased (*p* < 0.05). There were no statistically significant differences in MRI measurements between the no tear group and the supraspinatus tear group. CAL thickness was not statistically significant across all groups (Figure 6).

**Figure 7:**
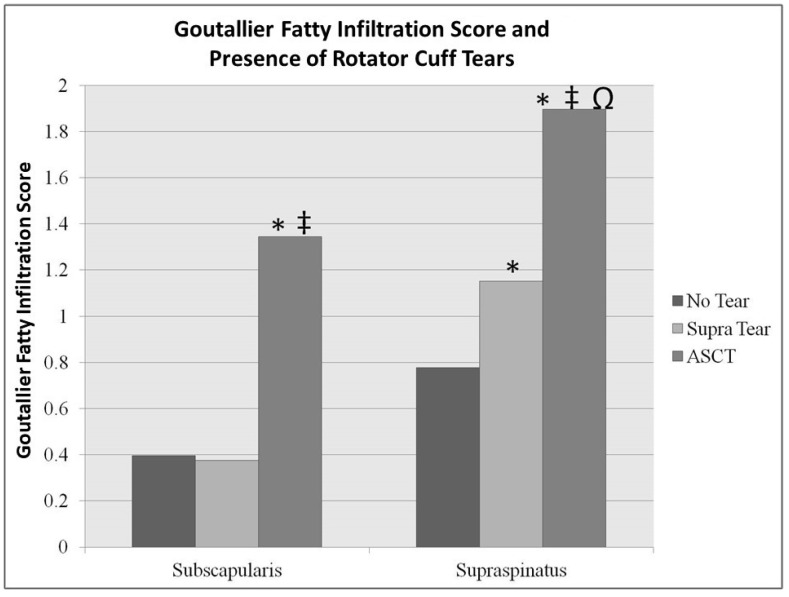
Goutallier fatty infiltration score in relation to rotator cuff tears. ASCT = Anterosuperior Cuff Tear. *, ‡ and Ω demonstrates statistically significant differences (*p* < 0.001) between supraspinatus tear vs no tear in supraspinatus group, between ASCT vs no tear, and ASCT vs supraspinatus tear.

The mean ± standard deviation (range) for each RC tear group Goutallier fatty infiltration classification is listed in Table 3. When comparing no tears to supraspinatus tears, the only significant MRI measurement identified was an increased fatty infiltration score in the supraspinatus tear group (1.15 ± 0.99) compared to the no tear group (0.78 ± 0.94) (*p* < 0.001). The supraspinatus fatty infiltration score was significantly higher in the ASCT when compared to isolated supraspinatus tears (*p* < 0.001) (Figure 7).

## 4. Discussion

To our knowledge, this study is one of the largest MRI-based analyses of quantitative measurements of coracoid impingement, and one of the first to qualitatively correlate the shape of the coracoid process with fatty infiltration and to associate RC tears in a non-cadaveric setting. The most significant findings of the present investigation were that several quantifiable MRI parameters consistent with subcoracoid impingement are associated with ASCT. In addition, the Goutallier fatty infiltration score was significantly higher in the ASCT group, indicating a relationship between fatty infiltration and ASCT.

Coracoid impingement can be a debilitating cause of anterior shoulder pain. Since the original description by Goldthwait [2], and further characterization by Gerber et al. [1], several studies aimed to elucidate the diagnosis of and treatment for coracoid impingement. However, the literature is controversial regarding the validity and reproducibility of using imaging methods to accurately diagnose this condition [30,31,32]. The present study is one of the largest non-cadaveric, MRI-based analyses of quantitative measurements of coracoid impingement, and one of the first to qualitatively associate the shape of the coracoid process with fatty infiltration and associated RC tears.

Our study had near equal distribution between male and female patients and between left and right shoulders. There were no sex or age-related differences in measurements of coracoid impingement. Giaroli et al. found a 3 mm difference between male and female subjects [31]. Females in our study had less than a 1mm difference in coracohumeral interval compared to male patients. While some sex adjustment may be necessary when measuring the coracohumeral interval, it may not be as large as previously reported. Age-related differences in coracoid morphology have been identified, suggesting that radiographic measurements may well yield different results that MRI-based measurements [9].

Previous research, classifying the coracoid process into morphological categories, based on whether its shape is flat, curved, or hooked found that a hooked coracoid was seen more frequently in tendinopathic and RC pathology [36]. The present study showed that a curved morphology was positively associated with the ASCT group, and a curved morphology was the most common coracoid shape in all patients. Specifically, there was a significantly greater number of curved coracoids than the hook or flat coracoids in the ASCT group compared to the no tear or isolated supraspinatus tear groups. However, previous research has indicated that the subcoracoid space becomes narrower and hooked in older shoulders, which possibly indicates a potential source of the above differing results [9]. Further research comparing specific age groups may be beneficial when comparing morphology and RC pathology.

Gerber et al. described coracoid impingement and developed some common measurements to evaluate possible impingement [1]. They referenced scapuloglenoid angle, CGA, CO, and CHI distance as useful measurements to relate the coracoid to the glenoid and humerus. As in the present investigation, they evidenced the wide inter-individual variability of the coracohumeral distance. A small CGA and a small CO were associated with a narrow subcoracoid space [1]. We used several measurements from Gerber et al. work [1]. In addition, we measured AHI, which was significantly associated with a coracoid impingement in patients with anterosuperior rotator cuff tears. We also found that coracoids with a larger CO and smaller CHI were significantly associated with ASCT.

In addition to comparing morphology and coracoid angle variabilities, our study uniquely also included a fatty infiltration score in the investigation of subcoracoid impingement. We found a significant difference in Goutallier fatty infiltration in both the subscapularis and supraspinatus tendons, with patients with anterosuperior rotator cuff tears having more atrophy than those with no tear, or isolated supraspinatus tear. Previous studies have reported that muscle atrophy and fatty infiltration, particularly of the infraspinatus, play a significant role in determining functional outcomes after cuff repair [21]. The significantly higher Goutallier grades in patients with anterosuperior rotator cuff tears may be an important determinant when considering surgical repair, as atrophy identification could optimize patient outcomes.

There are definite limitations to this study. For example, we did not study whether and how these imaging findings were associated with clinical and surgical findings. The addition of physical examination findings, as well as intraoperative findings, would be valuable in confirming the radiographic connection between coracoid impingement and anterosuperior rotator cuff tears. The position of the patient’s arm at the time of MRI can also affect the measurements between the coracoid and humerus. The arm was kept in neutral rotation in a shoulder coil, but even this standardization still likely resulted in a small amount of rotational difference among subjects. In addition, there was no inter-rater reliability test performed to ensure that there were no statistically different interpretations performed by the two MRI reviewers. Furthermore, the results of the ASCT group may have a selection bias as the age of these patients was significantly older than the two other groups, which may explain the higher fatty infiltration scores and subsequent RC tears [22,37] Future studies that better stratify study patients by age and correlate age and MRI measurements should be done in order to assess for this limitation. Lastly, the findings in this study are purely descriptive, and inferring causation rather than pure association with coracoid impingement, shoulder measurements, fatty infiltration, and RC tears would be beyond the scope of this study [38].

## 5. Conclusions

There is a wide variation in the relationship between the coracoid, glenoid, and humerus which does not appear to be sex or age-dependent. This study is unique in that it characterizes the shape of the coracoid and correlates the morphology with associated rotator cuff tears. Coracohumeral interval, acromiohumeral interval, and coracoid overlap are significantly altered in patients with associated anterosuperior rotator cuff tears. These measurements, in conjunction with the overall morphology of the coracoid, may assist in diagnosing and treating coracoid impingement.

## Figures and Tables

**Table 1:** Age, sex, and coracoid morphology for patients in each group. Values for age for each group are listed as Mean ± Standard deviation.

	No Tear (*n* = 63)	Supraspinatus Tear (*n* = 72)	Anterosuperior Rotator Cuff Tears (ASCT) (*n* = 58)
**Age**	52.2 ± 9.56 (40.3–87.2)	54.9 ± 8.76 (40.5–80)	60.3 ± 10.01 (42–87.6) *
**Sex**			
Male	28	37	37
Female	35	35	21
**Coracoid Morphology**			
Flat	5	9	1
Curved	34	42	46 *
Hooked	24	21	11

* Statistically significant compared to no tear and supraspinatus tear groups (*p* < 0.05).

**Table 2:** MRI measurements for each rotator cuff tear group. Values for each group are listed as Mean ± Standard deviation.

	No Tear (mm) (*n* = 63)	Supraspinatus Tear (mm) (*n* = 72)	Anterosuperior Rotator Cuff Tears (ASCT) (mm) (*n* = 58)	No Tear vs. Supraspinatus Tear	No Tear vs. ASCT	Supraspinatus Tear vs. ASCT
				*p*-Value	*p*-Value	*p*-Value
**Axial CR**	6.45 ± 1.72 (3.5–10.5)	6.42 ± 1.61 (1.8–10.2)	6.59 ± 1.7 (2.6–11)	0.920	0.668	0.577
**Axial CO**	12.87 ± 4.91 (2.1–22.1)	13.15 ± 4.78 (2.7–25.6)	14.92 ± 4.64 (5.1–24.8)	0.739	0.020 *	0.035 *
**Axial CHI**	10.83 ± 3.1 (6.1–20.6)	10.65 ± 3.33 (4.9–19.4)	9.14 ± 2.92 (2.8–16.1)	0.746	0.003 *	0.007 *
**Axial CGI**	20.04 ± 3.51 (13.2–27.4)	20.49 ± 3.06 (13.5–28.4)	21.3 ± 3.18 (15.1–28.7)	0.425	0.042 *	0.146
**CG angle**	146.79 ± 10.3 (127–176)	146.97 ± 11.52 (121–174)	143.14 ± 9.74 (126–167)	0.925	0.048 *	0.046 *
**Sag CHI**	11.173 ± 3.25 (5.9–24.4)	10.9 ± 3.31 (4.1–19.6)	9.47 ± 2.63 (2.6–16.1)	0.625	0.002 *	0.009 *
**Sag CAL** **thickness**	1.78 ± 0.66 (0.8–4.1)	1.92 ± 0.69 (0.9–3.7)	2.03 ± 0.75 (0.9–4.6)	0.224	0.053	0.389
**Cor AHI**	6.54 ± 1.29 (2–9.7)	6.53 ± 1.37 (3.9–11.4)	5.69 ± 1.84 (1.4–8.9)	0.978	0.004 *	0.003 *

* Statistically significant compared to no tear and supraspinatus tear groups (*p* < 0.05).

**Table 3:** Goutallier fatty infiltration classification for each rotator cuff tear group. Values for each group are listed as Mean ± Standard deviation.

	No Tear (mm) (*n* = 63)	Supraspinatus Tear (mm) (*n* = 72)	Anterosuperior Rotator Cuff Tears (ASCT) (mm) (*n* = 58)	No Tear vs. Supraspinatus Tear	No Tear vs. ASCT	Supraspinatus Tear vs. ASCT
				*p*-Value	*p*-Value	*p*-Value
**Goutallier Subscap**	0.4 ± 0.75 (0–3)	0.37 ± 0.54 (0–2)	1.34 ± 0.91 (0–3)	0.846	0.001 *	0.001 *
**Goutallier Supra**	0.78 ± 0.94 (0–4)	1.15 ± 0.99 (0–4)	1.9 ± 1.24 (0–4)	0.026 *	0.001 *	0.001 *

* Statistically significant compared to no tear and supraspinatus tear groups (*p* < 0.001).

## Data Availability

Not applicable.
